# MicroRNA-222 Expression and Its Prognostic Potential in Non-Small Cell Lung Cancer

**DOI:** 10.1155/2014/908326

**Published:** 2014-04-13

**Authors:** Kai-ping Mao, Wei-na Zhang, Xiao-min Liang, Yu-rong Ma

**Affiliations:** Department of Thoracic Surgery, Qingdao Municipal Hospital, No. 5 Donghai Middle Road, Qingdao 266071, China

## Abstract

Overexpression of miR-222 has been found in several types of cancers; however, the expression of miR-222 in non-small cell lung cancer (NSCLC) and its prognostic values are unclear. This study aimed to investigate whether the miR-222 expression level is related to clinicopathological factors and prognosis of NSCLC. Through a prospective study, 100 pairs of NSCLC tissues and adjacent normal tissues were examined by quantitative reverse-transcription polymerase chain reaction. The correlation between miR-222 expression and clinicopathological features was analyzed, and the significance of miR-222 as a prognostic factor and its relationship with survival were determined. Results showed that the expression levels of miR-222 were significantly elevated in the NSCLC tissue compared with that in adjacent normal tissue. In addition, Cox's proportional hazards model analysis confirmed that miR-222 high expression level was an independent predictor of poor prognosis. In conclusion, miR-222 overexpression is involved in the poor prognosis of NSCLC and can be used as a biomarker for selection of cases requiring especial attention.

## 1. Introduction

Lung cancer, predominantly non-small-cell lung cancer (NSCLC), is the leading cause of cancer-related mortality worldwide, with 5-year survival as low as 13% [1]. NSCLC includes two predominant subtypes, adenocarcinoma and squamous cell carcinoma, which comprise 40 and 25%, respectively [2]. Unfortunately, local and/or distant metastases have developed in up to 75% of the lung cancer patients when clinically diagnosed [3]. Thus, there is a need to identify new, noninvasive prognostic biomarkers for NSCLC in order to improve postoperative treatment strategies.

MicroRNAs (miRNAs) are small, noncoding RNAs that regulate the translation of specific protein coding genes. Mature miRNAs are small (20-21 nucleotides in length) endogenous noncoding RNAs that regulate the expression of target genes at the posttranscriptional level through degradation of transcripts and inhibition of translation by mainly binding to 3′-UTR of target messenger RNA (mRNA) [4]. Recent studies have revealed the role of miRNAs in a variety of basic biological and pathological processes [5], and the association of miRNA signatures with human diseases has been established [6, 7]. Recently, accumulating evidence has demonstrated that microRNA-222 (miR-222) plays a crucial role in cancer cell proliferation [8], and overexpression of miR-222 has been found in several types of cancers such as breast cancer, bladder cancer, colorectal carcinoma, glioblastoma, and pancreatic cancer [9–13]. However, the expression of miR-222 in NSCLC and its prognostic values still remain unclear.

The aim of the present study is to evaluate the clinical significance of miR-222. The expression level of the miR-222 was measured in both adjacent normal tissue and cancerous tissue. Furthermore, the correlation between the expression level of miR-222 and clinicopathological characters was analyzed. In addition, the influence of miR-222 on the prognosis of NSCLC patients was estimated.

## 2. Materials and Methods

### 2.1. Patients and Samples

This study was approved by the Research Ethics Committee of Qingdao Municipal Hospital. Written informed consent was obtained from all of the patients. All specimens were handled and made anonymous according to the ethical and legal standards. The selection criteria for patients with NSCLC were as follows: (1) pathologically confirmed patients with NSCLC and (2) the patients who had no previous history of other cancers. All patients were diagnosed and treated at the Qingdao Municipal Hospital in Shandong, China, from November 2007 to June 2012. All subjects underwent clinical examination; plain chest radiograph; CT scan of the chest, upper abdomen, and brain; fiberoptic bronchoscopy; and bone scan. Tumor stage was determined according to the 2009 TNM staging classification system. The duration of follow-up was calculated from the date of surgery to death or last follow-up, and patients were excluded if they had incomplete medical records or inadequate follow-up. For qRT-PCR, 100 pairs of fresh NSCLC and matched adjacent normal tissue specimens were collected from patients who underwent surgery in the Qingdao Municipal Hospital. The fresh tissue specimens were collected and immediately placed in liquid nitrogen and then stored at −80°C until the isolation of RNA. Clinicopathological features of patients are summarized in Table 1.

### 2.2. MicroRNA Isolation and Real-Time Quantitative RT-PCR Assay

Total RNA was isolated from frozen specimen by homogenizing tissue in Trizol reagent (Invitrogen, Carlsbad, CA, USA), according to the manufacturer's instructions. The purity and concentration of RNA were determined using NanoDrop 1000 spectrophotometer (Thermo Scientific, Wilmington, DE, USA). The differentially expressed amount of the miR-222 was validated in triplicate by quantitative reverse-transcription polymerase chain reaction (qRT-PCR). Briefly, 2 *μ*g of RNA was added to RT reaction, and then, the cDNA served as the template for amplification of PCR with sequence-specific primers (Sangon Biotech, Shanghai, China) using SYBR PrimeScript miRNA RT-PCR kit (Takara Biotechnology Co., Ltd., Dalian, China) on the 7500 Real-Time PCR systems (Applied Biosystems, Carlsbad, CA, USA). The PCR cycling profile was denatured at 95°C for 30 s, followed by 40 cycles of annealing at 95°C for 5 s and extension at 60°C for 34 s. Small nucleolar RNA U6 was used as an internal standard for normalization. The cycle threshold (*C*_*T*_) value was calculated. The 2^−Δ*C*_*T*_^  (Δ*C*_*T*_ = *C*_*T* miR-222_ − *C*_*T* U6  RNA_) method was used to quantify relative amount of miR-222.

### 2.3. Statistical Analysis

The comparison of the expression level of miR-222 between NSCLC tissue and adjacent normal tissue was performed using two-sample Student's *t*-test. The correlation between the expression of miR-222 and clinicopathological characters was assessed with two-sample Student's *t*-test. The overall survival was analyzed by log-rank test, and survival curves were plotted according to Kaplan-Meier. The univariate Cox regression was performed on each clinical covariate to examine its influence on patient survival. Final multivariate models were based on stepwise addition. A Wald statistic of  *P* < 0.05 was used as the criterion for inclusion in final multivariate models. All tests were two tailed and results with *P* < 0.05 were considered statistically significant. Statistical analyses were performed using SPSS 13.0 software (Chicago, IL, USA) and GraphPad Prism 5 (GraphPad Software Inc., CA, USA).

## 3. Results

### 3.1. Expression of miR-222 in NSCLC Tissues by qRT-PCR

**Figure 1:**
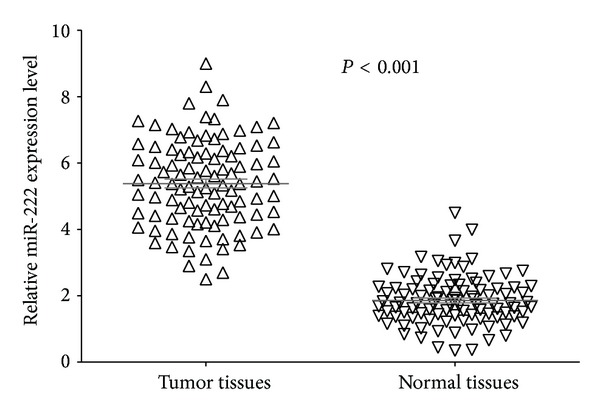
Comparison of miR-222 expression levels between NSCLC tissue and adjacent normal tissue. Analysis using two-sample Student's *t*-test showed that the relative expression levels of miR-222 in the NSCLC tissue were significantly higher than those in adjacent normal tissue (*P* < 0.001).

We examined miR-222 expression in 100 pairs of NSCLC tissues and the corresponding noncancerous tissues by qRT-PCR. As shown in Figure 1, the expression level of miR-222 was significantly higher in tumor tissues than in corresponding noncancerous tissues (5.37 ± 1.36 versus 1.77 ± 0.79, *P* < 0.001).

### 3.2. Relation of miR-222 Expression Level to Clinicopathological Characteristics

Table 1 presents the results of the correlation analysis between the relative miR-222 expression levels and clinicopathological features of NSCLC. There was no correlation between the relative miR-222 expression levels and age and sex, but the relative miR-222 expression levels were significantly positively correlated with TNM stage and regional lymph node involvement. The relative miR-222 expression level was significantly higher in patients with stage III NSCLC compared with patients with stage I-II disease (*P* < 0.001) and in patients with lymph node involvement compared with patients without regional lymph node involvement (*P* < 0.001).

### 3.3. Expression of miR-222 from Patients with NSCLC in relation to Prognosis

**Figure 2:**
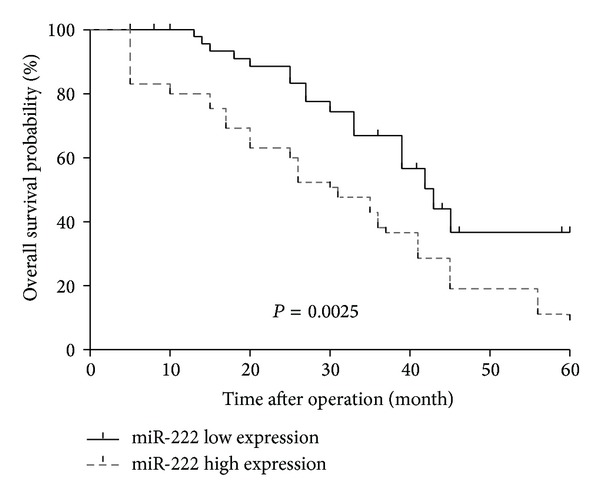
The Kaplan-Meier survival curve in relation to miR-222 expression level in 100 patients with NSCLC. The survival rate of patients with high miR-222 level was significantly lower than that of patients with low miR-222 level (log-rank test *P* = 0.0025).

To evaluate whether miR-222 expression levels can predict NSCLC prognosis, we next performed survival analysis. The miR-222 expression level was classified as high or low in relation to the median value. The Kaplan-Meier analysis showed that patients with higher levels of miR-222 had significantly poorer survival than those with lower expression of this miRNA in patients with 5-year overall survival of 9.37% and 36.69%, respectively (*P* = 0.0025; Figure 2).

A Cox proportional hazards analysis was used to further evaluate the potential of miR-222 expression as a prognostic biomarker. Univariate survival analyses indicated that miR-222 expression, TNM stage, and regional lymph node involvement were associated with prognosis, while gender and age were not associated with prognosis. In the multivariate Cox proportional hazards analysis, which included miR-222, TNM stage, and regional lymph node involvement, high miR-222 expression was independently associated with poor survival (*P* < 0.001; HR = 3.31; 95% CI = 1.97–5.58; Table 2).

## 4. Discussion

Identification of miRNA molecular profiles associated with the prognosis of patients with NSCLC may not only elucidate the underlying biological mechanisms involved in the development or progression of the disease but also provide the opportunity to identify novel targets for NSCLC therapy. In 2011, Zhang et al. found that miR-222 was upregulated in NSCLC samples (*P* = 0.00032) [14]. However, the clinical significance of miR-222 gene expression in NSCLC remains unclear. In the present study, we found that miR-222 expression was proven to be associated with advanced clinical stage and lymph node metastases, suggesting that miR-222 might be involved in the carcinogenesis and metastasis of NSCLC. More importantly, we proved that patients with a high expression of miR-222 tended to have shorter survival than patients with lower levels, indicating that high miR-222 level is a marker of poor prognosis for patients with NSCLC.

Overexpression of miR-222 has been observed in several types of cancers, suggesting its important role in tumorigenesis. Lee et al. found that the tissue expression levels of miR-222 were significantly upregulated in pancreatic cancer samples compared with those in adjacent normal tissues (*P* = 0.00001) [10]. Multivariate analysis with Cox's proportional hazards model confirmed that the miR-222 high expression level was an independent predictor of poor prognosis for the patients with pancreatic cancer. Furthermore, they found that expression of miR-222 in the tissues was strongly related to the expression levels of Ki67, which was strictly related to cell proliferation [15]. Puerta-Gil et al. found that the expression level of miR-222 was significantly higher in bladder cancer tissues than in corresponding noncancerous tissues [11]. In addition, miR-222 expression was significantly correlated with increasing tumor grade (*P* = 0.017), tumor size (*P* = 0.005), presence of carcinoma in situ (*P* = 0.035), and clinical outcome end points (recurrence, *P* = 0.006; progression, *P* = 0.003; disease specific, *P* = 0.034; and overall survival, *P* = 0.023). In agreement with these studies, we confirmed that the expression levels of miR-222 in NSCLC tissues were significantly higher than those in normal adjacent tissue, and high miR-222 expression was independently associated with poor survival. So miR-222 may be involved in NSCLC progression.

The molecular mechanisms behind the altered expression of miR-222 in NSCLC have been investigated by some researchers. Zhang et al. found that the expression of high-mobility group A1 (HMGA1) was in positive correlation with miR-222 in NSCLC samples [14]. Then they investigated whether HMGA1 was responsible for the enhanced expression of oncogenic miR-222 in NSCLC cells. Chromatin immunoprecipitation (CHIP) assay revealed that HMGA1 directly bound to the proximal promoter of miR-222 in NSCLC cells. HMGA1 silencing reduced miR-222 transcriptional activity, whereas forced HMGA1 expression increased it, indicating that miR-222 was directly regulated by HMGA1. In addition, they found that the overexpression of miR-222 in NSCLC could lead to repression of phosphatase 2A subunit B (PPP2R2A) expression and activation of Akt signaling. Acunzo et al. and Garofalo et al. found that miR-130a, expressed at low level in lung cancer cell lines, by targeting MET was able to reduce TNF-related apoptosis-inducing ligand (TRAIL) resistance in NSCLC cells through the c-Jun-mediated downregulation of miR-221 and miR-222 [16, 17]. So, miR-222 has the potential to become a promising therapeutic target or diagnostic tool for TRAIL resistance in NSCLC. Furthermore, miR-222 has been found to target p27, p57, and PTEN expression to promote cell-cycle progression [18–20]. Recently, Wang et al. found that metformin inhibited lung cancer cells proliferation through repressing miR-222 [21].

In conclusion, our results showed that miR-222 was overexpressed in NSCLC, and its high expression indicated an association with poor prognostic factors and poor survival. To clarify the role of miR-222 as well as its use as a biomarker and in targeting therapy, large worldwide population-based studies with a standard definition of miR-222 expression level are necessary.

## Figures and Tables

**Table 1:** Correlation between miR-222 expression and clinicopathological characters in 100 non-small cell lung cancer patients.

Variables	*n*	Relative miR-222 level	*P* value
Age, y			
<60	34	5.32 ± 0.77	
≥60	66	5.44 ± 0.51	0.76
Sex distribution			
Female	41	5.11 ± 0.76	
Male	59	5.47 ± 0.61	0.83
TNM stage			
I-II	43	4.19 ± 0.31	
III	57	6.21 ± 0.46	<0.001
Regional lymph node involvement			
Absent	47	4.39 ± 0.49	
Present	53	6.33 ± 0.94	<0.001

**Table 2:** Multivariate analyses for prognostic factors in patients with non-small cell lung cancer.

Variables	Hazard ratio (95% CI)	*P* value
Age (<60 versus ≥60)	0.98 (0.81–1.39)	0.11
Sex distribution (male versus female)	1.31 (0.77–1.89)	0.17
TNM stage (I-II versus III)	0.76 (0.65–0.89)	0.009
Regional lymph node involvement (absent versus present)	0.68 (0.39–0.77)	0.007
miR-222 (high versus low)	3.31 (1.97–5.58)	<0.001

CI: confidence interval.
